# Ancient home or in exile? The easternmost species of genus *Starengovia* Snegovaya, 2010 found in China (Opiliones, Nemastomatidae, Nemastomatinae)

**DOI:** 10.3897/zookeys.770.25491

**Published:** 2018-07-04

**Authors:** Chao Zhang, Jochen Martens

**Affiliations:** 1 The Key Laboratory of Invertebrate Systematics and Application, College of Life Sciences, Hebei University, Baoding, Hebei 071002, China; 2 Institut für Organismische und Molekulare Evolutionsbiologie, D-55099 Mainz, Germany; 3 Senckenberg Research Institute, Arachnology. Frankfurt am Main, Germany

**Keywords:** East Palaearctic, genitalia, harvestmen, relict species, *Starengovia*, taxonomy, Yunnan Province

## Abstract

*Starengovia quadrituberculata* **sp. n.** is described and illustrated based on male and female specimens collected in Yunnan Province, China. The new species is distinct from the two other congeners, *S. kirgizica* Snegovaya, 2010 and *S. ivanloebli* Martens, 2017, in having two pairs of low submedian tubercles on abdominal areae III and IV; distal margin of the lateral foliate wing-like structures of the penis situated close to the glans base, the short rod-like stylus, the form and position of spines on the stylus of the penis, anvil-shaped tubercles mainly on front margin of prosoma. The occurrence of *Starengovia* in Yunnan, the second nemastomatine species in China, creates a huge distributional gap of roughly 2700 km distance to its closest neighbor *S. ivanloebli* in Northwest Pakistan. The historical relations of Chinese nemastomatines are discussed.

## Introduction

The family Nemastomatidae Simon, 1872 is currently represented by two subfamilies (Ortholasmatinae Shear & Gruber, 1983, and Nemastomatinae Simon, 1872) and includes 23 genera and 138 species worldwide (Schönhofer 2013; Zhang and Zhang 2013; Martens 2006, 2016, 2017; Zhang, Zhao and Zhang 2018). Distribution of Nemastomatinae is predominantly West Palaearctic covering nearly all parts of Europe, and beyond Europe penetrating, e.g., to Kyrgyzstan and in the Pamir Mts. Recently, the first nemastomatine harvestman was discovered in China and assigned to a new genus, *Sinostoma* Martens, 2016, extending the distribution of nemastomatines to approximately 3000 km southeastwards. Here another minute nemastomatine harvestman species is described from China in the mountains of southern Yunnan Province.

## Materials and methods

Taxonomic scheme follow the outline proposed by Gruber (2007). The specimens were preserved in 75% ethanol, examined, and drawn under a Leica M205A stereomicroscope equipped with a drawing tube. Photographs were taken using a Leica M205A stereomicroscope equipped with a DFC 450 CCD. The type specimens are deposited in the Museum of Hebei University, Baoding, China (MHBU). All measurements are given in mm.

## Taxonomy

### Nemastomatidae Simon, 1872

#### Nemastomatinae Simon, 1872

##### Starengovia

Taxon classificationAnimaliaOpilionesNemastomatidae

Snegovaya, 2010

Starengovia Snegovaya, 2010: 351–352; Schönhofer 2013: 47; Martens 2017: 187–188.

###### Type species.

*Starengovia kirgizica* Snegovaya, 2010, original designation.

###### Diagnosis.

Small species up to 1.7 mm, dorsal scutum with lines of anvil-shaped tubercles along margins of scutal areas. Pairs of para-median tubercles on opisthosomal areas of dorsal scutum. Truncus penis moderately slender, large muscle-containing inflated base, truncus in straight continuation of inflated base. Distal part of truncus with one large lateral wing on either side, glans inconspicuous, not well differentiated from truncus; armament of glans simple with symmetrical arrangement. Apophysis on basal cheliceral article of male well-marked, with a distad-directed hook, discharge area for secretion in a bowl-like excavation on medial side of apophysis (Martens 2017).

###### Distribution.

China (Yunnan), Kyrgyzstan, Uzbekistan, Himalayas of Pakistan.

###### Key to the currently known species of *Starengovia*

**Table:** 

1	Distributed in Yunnan, China, low para-median tubercles on opisthosomal areae III and IV (Figs 1, 8, 13, 26–27, 29–30), penis with alae of wings bent to ventral side (Figs 20–22)	***S. quadrituberculata* sp. n.**
–	Distributed in Central Asia (Kyrgyzstan, Uzbekistan) and NW Pakistan; high slender or compact para-median tubercles on opisthosomal areae I–V; penis with alae of wings bent to ventral side or straight, not bent	**2**
2	Distributed in Kyrgyzstan (one record also in Uzbekistan), tubercles of dorsal scutum conical and compact; penis with alae of wings bent to ventral side	***S. kirgizica***
–	Distributed in northwestern Pakistan, tubercles of dorsal scutum slender, penis with alae of wings straight, not bent to ventral side	***S. ivanloebli***

##### Starengovia quadrituberculata sp. n.

Taxon classificationAnimaliaOpilionesNemastomatidae

http://zoobank.org/3A5FA717-6819-4873-BBE4-F14974B8F879

Figs 1–7 , 8–19 , 20–25 , 26–31

###### Diagnosis.

Areae III–IV of opisthosomal region each with a pair of very low median tubercles inclined posteriorly. Basal segment of chelicerae dorso-distally with a triangular apophysis in male (in lateral view). Distal part of penis with extended lateral wing structure; width of the wings almost equivalent to length. Glans short, nearly cone-shaped; stylus short and conical. Scanty anvil-shaped tubercles confined to front margin of prosoma.

###### Type locality.

CHINA, Yunnan Province: Baoshan City, Lujiang Town, Dahaoping, 24°57'42"N, 98°43'58"E, 2142 m ASL, evergreen forest, sifted from leaf litter.

**Type specimen.** Holotype male (MHBU-Opi-20171208). Adult male preserved in 75% ethanol, with genitalia in a separate microvial. Original label: MHBU-Opi-20171208, CHINA: Yunnan Province, Baoshan City, Lujiang Town, Dahaoping, 24°57'42"N, 98°43'58"E, 2142 m ASL, 23 November 2017, Y.N. Mu leg.

###### Paratype.

1♀ (MHBU-Opi-20171209), same data as the holotype.

###### Etymology.

The specific name is taken from the Latin *quadri*- (four) and *tuberculum* (tubercle, small apophysis), referring to the two pairs of small tubercles on opisthosomal areae III and IV.

###### Description of the male holotype.

**Figures 1–7.**
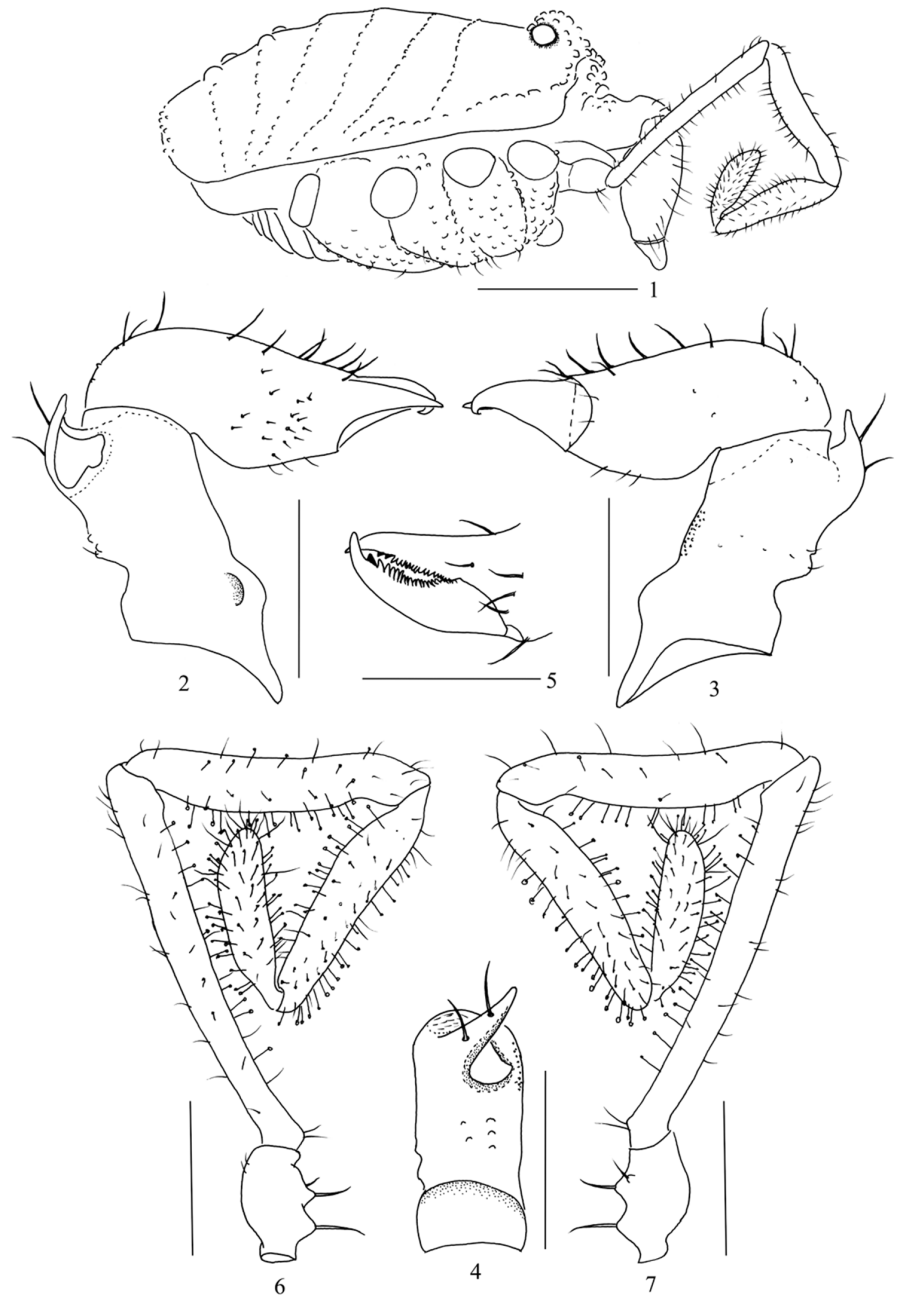
*Starengovia quadrituberculata* sp. n. male (holotype) **1** Body, lateral view **2** Left chelicera, medial view **3** Left chelicera, ectal view **4** Basal segment of left chelicera, dorsal view **5** Left cheliceral fingers, frontal view **6** Left pedipalp, medial view **7** Left pedipalp, ectal view. Scale bars 0.5 mm (**1**); 0.25 mm (**2–7**).

**Figures 8–19.**
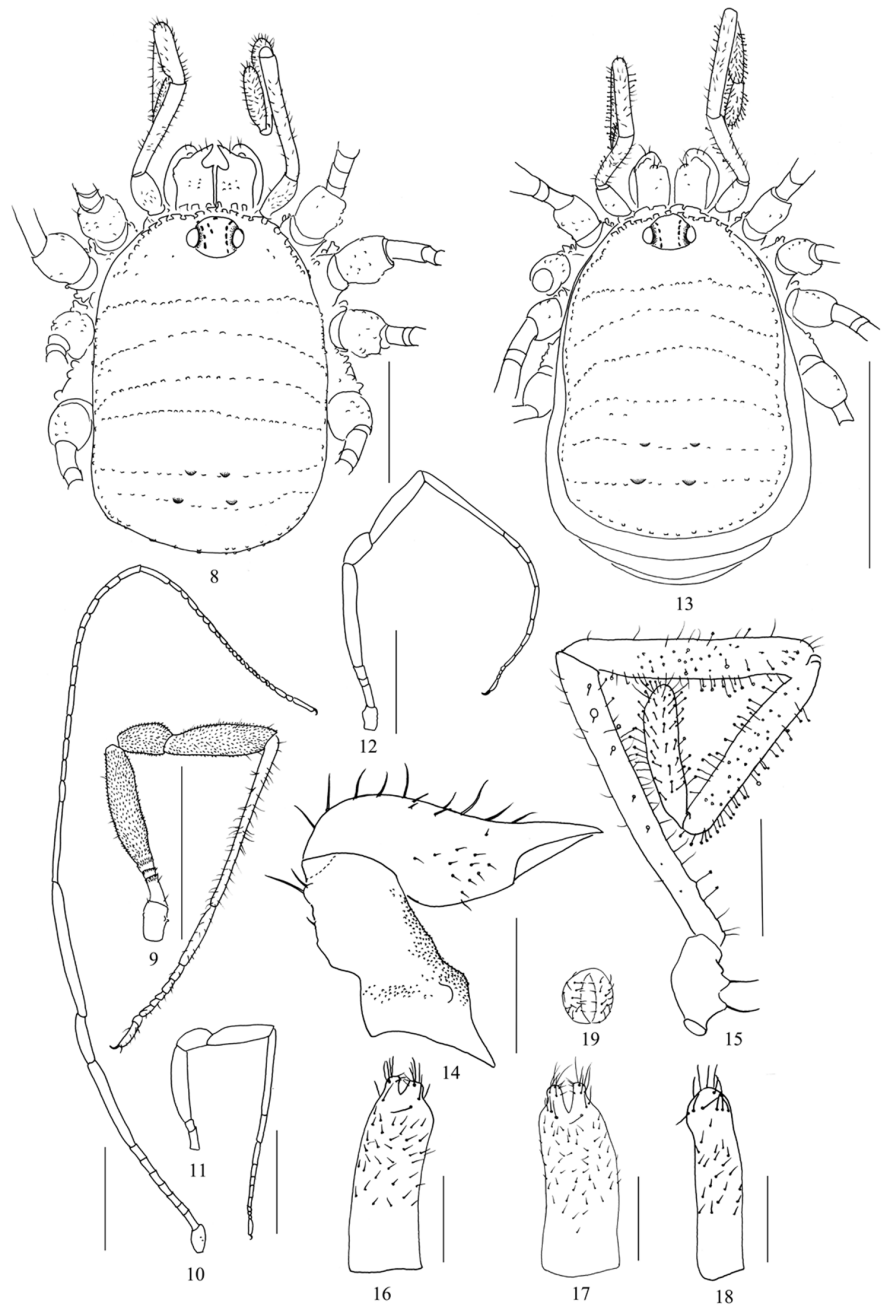
*Starengovia quadrituberculata* sp. n. **8** Body, male, dorsal view **9–12** Right legs, retrolateral view **9** Leg I **10** Leg II **11** Leg III **12** Leg IV **13** Body, female, dorsal view **14** Left chelicera, female, medial view **15** Left pedipalp, female, medial view **16** Ovipositor, ventral view **17** Ovipositor, dorsal view **18** Ovipositor, lateral view **19** Ovipositor, frontal view. Scale bars 1 mm (**9–13**); 0.5 mm (**8**); 0.25 mm (**14–18**).

Habitus as in Figs 1, 8, 26–28. Coloration in alcohol: dorsum brown black, without silvery or golden markings (Fig. 26). Venter concolorous with the dorsum (Fig. 28), but intersegmental membranes whitish. Chelicerae and pedipalpi chestnut-brown. Legs deep black.

**Dorsum** (Figs 8, 26). Body small, strongly sclerotized. Dorsal scutum ovoid in shape. Anterior margin of the carapace nearly rounded, armed with a continuous row of anvil-shaped tubercles, posterior margin slightly rounded, more quadrangular. Ocularium slightly elevated, rising from frontal margin of scutum, irregularly covered with quadrangular tubercles. Supracheliceral lamellae consisting of three small sclerite plates. Metapeltidial area and opisthosomal region (areae I–V) separated by lines of quadrangular tubercles similar to those at peripheral margins of the scutum. Areae III–IV each with a pair of low median pegs inclined posteriorly. Free tergites not visible from above.

**Venter** (Fig. 28). Coxae with scattered low rounded tubercles on ventral surfaces and pro-laterally and retro-laterally with a row of quadrangular tubercles. Genital operculum short, almost tongue-shaped, surface with scattered tubercles. Free sternites with few tubercles at margins.

**Chelicerae** (Figs 2–5). Basal segment ventrally and medially each with a rounded hump at the base (medial view, Fig. 2), and dorso-distally with a triangular apophysis distinctly surpassing front margin of basal segment, approximately as long as high (in lateral view), medially compressed and spoon-shaped (medial view, Fig. 2); apophysis medially inclined dorso-distally projecting into a pointed hook, dorsally with two long setae; the medial excavation of apophysis harbouring the secretion porefield; a few tubercles laterally and dorsally on medial part of basal segment (Figs 3–4); a multitude of minute granules on the ventro-lateral surface of basal segment (Fig. 3). The second segment with a few tubercles laterally and dorsally at base. Many long dorsal setae and rows of short setae at base of fixed finger (Fig. 2). Fingers short, with diaphanous teeth and dark subapical teeth: one dark tooth on movable finger, two dark teeth on fixed finger (Fig. 5).

**Pedipalpi** (Figs 6–7). Trochanters with three ventral seta-tipped tubercles. Femora and patellae with normal straight setae mainly on dorsal and lateral sides. Femora slightly swollen distally and ventrally with sparse clavate setae. Patellae ventrally slightly thickened and medially, ventrally and laterally with sparse clavate setae. Tibiae and tarsi densely covered with clavate setae all round.

**Legs** (Figs 9–12). Femora, patellae, and tibiae of leg I, III and IV slightly inflated. Femora, patellae, and tibiae of all legs densely covered with stiff, short bristles (Fig. 9). Pseudoarticulations of femora I–IV: 2/7/2/3; pseudoarticulations of metatarsi I–IV: 0/13/1/3. Tarsal segments I–II with two tarsomere groups: 8 (6+2), 24 (22+2); III–IV with three each: 9 (5+2+2), 9 (5+2+2).

**Figures 20–25.**
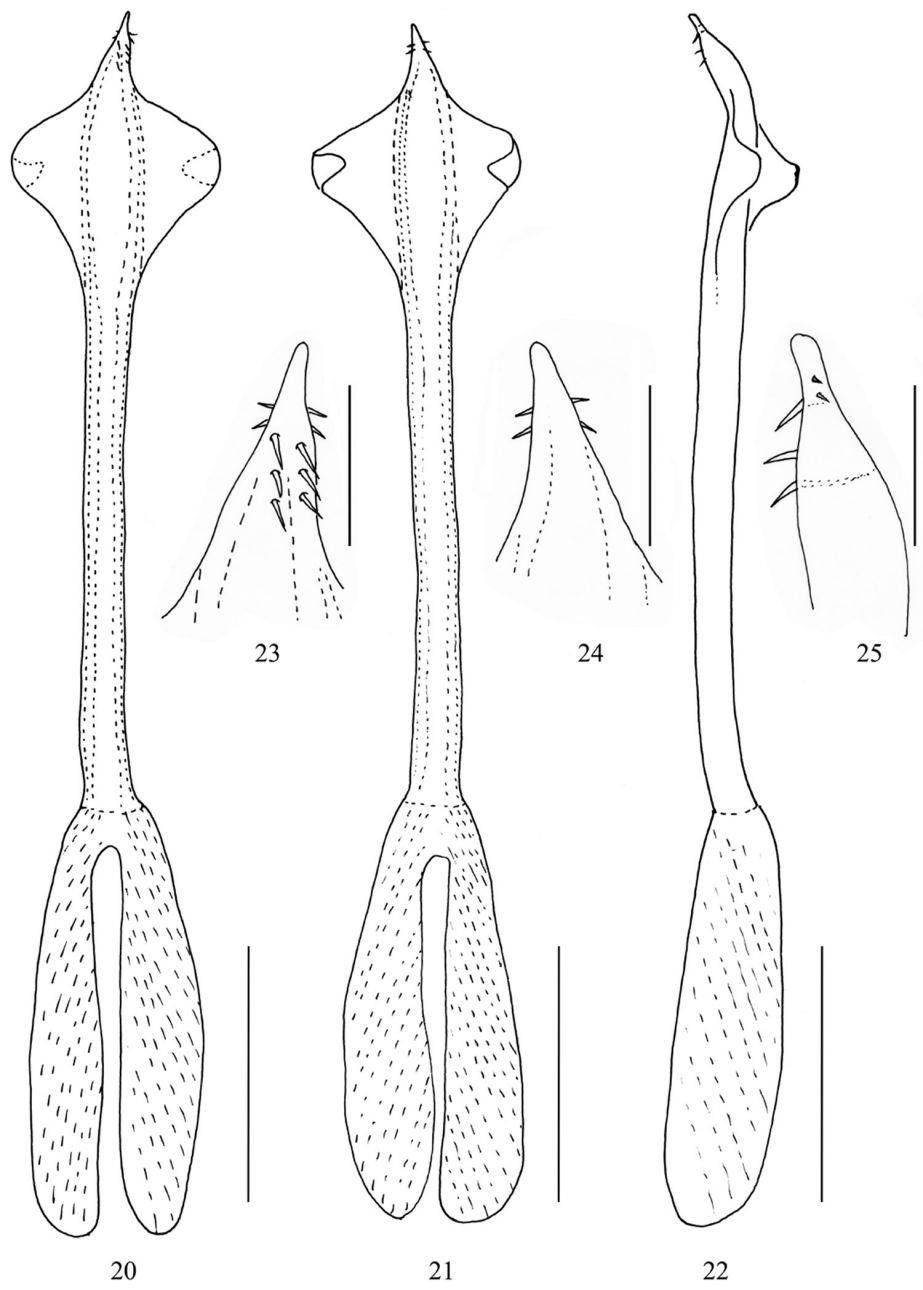
*Starengovia quadrituberculata* sp. n. male (holotype) **20** Penis, dorsal view **21** Penis, ventral view **22** Penis, lateral view **23** Penis tip, dorsal view **24** Penis tip, ventral view **25** Penis tip, lateral view. Scale bars 0.25 mm (**20–22**); 0.05 mm (**23–25**).

**Penis** (Figs 20–25). Moderately slender; no clear distinction between truncus, glans, and stylus. Basis forming a large inflated part (occupying approximately one third of whole penis length) and deeply split into two parts each bearing one large muscle portions, basis well differentiated from rest of truncus; truncus beyond basis parallel-sided, distal portion close to glans inconspicuously curved (lateral view). Ventro-lateral side of truncus sub-distally with two broad foliate wing-like structures forming a transparent membrane, triangular, free pointed end curled to ventral side. Glans extremely short, armament of glans with pairs of short spicule-like setae; three pairs on dorsal side, two pairs more distally on both “lateral” sides, stylus short and rod-like.

**Figures 26–31.**
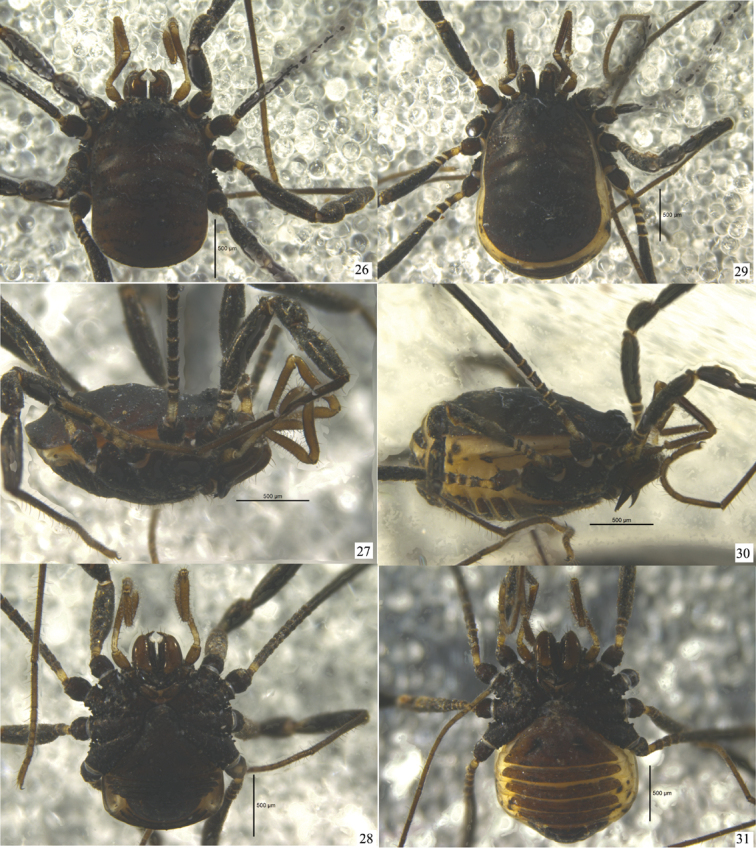
*Starengovia quadrituberculata* sp. n. Photographs of holotype male and female paratype **26** Body and parts of appendages, male, dorsal view **27** Ditto, lateral view **28** Ditto, ventral view **29** Body and parts of appendages, female, dorsal view **30** Ditto, lateral view **31** Ditto, ventral view. Scale bars 0.5 mm.

**Female** (Figs 13–15, 29–31). In appearance and coloration similar to the male, but body much larger (Figs 13, 29). Free tergites visible from above (Fig. 13). Basal segment of chelicerae dorso-distally with a hump covered with two long setae, and ventrally with a multitude of minute granules, similar granules medially at the base (Fig. 14). Patellae of pedipalpi with many clavate hairs (Fig. 15). Pseudoarticulations of femora I–IV: 2/7/2/3–4; pseudoarticulations of metatarsi I–IV: 1/14/2/3. Tarsal segments I–IV: 9 (7+2), 15 (13+2), 8 (4+2+2), 10 (6+2+2).

**Ovipositor** (Figs 16–19). Short type (Martens et al. 1981, Suzuki 1974: 88), unsegmented. The apical furca bipartite, each bearing 16 setae in three groups: six long setae at the base of furca (Fig. 18), four short ones medially at the margin of apical lobe (Fig. 19), and six long ones between former two groups (Fig. 19).

###### Measurements.

Male holotype (female paratype): Body 1.41 (1.75) long. 0.96 (1.26) wide at the widest portion. Ocularium 0.13 (0.18) long, 0.23 (0.23) wide. Basal segment of chelicerae 0.32 (0.30) long; second segment of chelicerae 0.45 (0.54) long. Penis 0.75 long (including glans), 0.05 wide at base, alate part 0.20 wide, fork 0.39 long. Ovipositor 0.60 long. Measurements of left pedipalp and right legs as in Tables 1, 2.

**Table 1.** *Starengovia quadrituberculata* sp. n. Measurements of the pedipalp and legs of the male holotype, as length/depth.

	Trochanter	Femur	Patella	Tibia	Metatarsus	Tarsus	Total
Pedipalp	0.21/0.11	0.63/0.06	0.48/0.07	0.41/0.08		0.27/0.07	2.00
Leg I	0.22/0.14	0.91/0.16	0.37/0.17	0.59/0.17	1.15/0.06	1.01/0.05	4.25
Leg II	0.22/0.14	1.85/0.09	0.50/0.13	1.28/0.10	3.29/0.06	2.17/0.05	9.31
Leg III	0.22/0.14	1.01/0.16	0.34/0.18	0.56/0.14	1.13/0.06	0.96/0.05	4.22
Leg IV	0.22/0.14	1.40/0.14	0.36/0.17	0.79/0.15	1.65/0.06	1.20/0.05	5.62

**Table 2.** *Starengovia quadrituberculata* sp. n. Measurements of the pedipalp and legs of the female paratype, as length/depth.

	Trochanter	Femur	Patella	Tibia	Metatarsus	Tarsus	Total
Pedipalp	0.23/0.11	0.66/0.06	0.53/0.07	0.42/0.06		0.29/0.07	2.13
Leg I	0.22/0.14	0.89/0.15	0.37/0.16	0.59/0.15	1.13/0.06	1.02/0.05	4.22
Leg II	0.22/0.14	1.82/0.09	0.53/0.13	1.22/0.10	3.26/0.06	2.13/0.05	9.18
Leg III	0.22/0.14	0.91/0.14	0.35/0.17	0.53/0.14	1.15/0.06	0.91/0.05	4.07
Leg IV	0.22/0.14	1.36/0.13	0.37/0.17	0.70/0.12	1.60/0.06	1.19/0.05	5.44

###### Habitat.

The specimens were collected by leaf litter sieving in broad-leaved forest under dense canopy at an altitude of 2142 m ASL.

###### Distribution.

Known only from the type locality in southern Yunnan Province, China.

## Discussion

The discovery of a species of the genus *Starengovia* in Yunnan comes quite unexpectedly. *Starengovia* is known from Central Asian Kyrgizstan and Uzbekistan only by a few localized records of *S. kirgizica* Snegovaya, 2010. *Starengovia ivanloebli* Martens, 2017 is known from the Himalayas of Northwest Pakistan, disjunct by 700 km. The present record of *S. quadrituberculata* sp. n. moves the distributional limit of nemastomatines by a second species by roughly 2700 km to the Southeast to southern Yunnan Province in China. The first nemastomatine ever discovered in China is *Sinostoma yunnanicum* Martens, 2016, only 380 km to the northeast of the present record, in Yunnan as well. Both are minute species less than 2 mm in body length, difficult to discover and apparently restricted to primeval mountain forests above 2000 m.

The few records of nemastomatines in East Asia hitherto known are restricted to two genera and appear to be remarkably disjunct from the European nemastomatine core distributional area. Though more local Asian occurrences may be discovered in the future, these are rare harvestmen and probably relicts of old lineages which do not exist in the West Palaearctic and probably never occurred there. According to only punctual, disjunct distributional areas and morphological traits Central Asian and Chinese occurrences of nemastomatines seem to represent “ancient homes” rather than “recent exiles”, i.e., geographical outliers of the main prosperous and speciose West Palaearctic radiations. This is meant in a dynamic sense – not just static as marginal occurrences.

In accordance with this hypothesis, *Starengovia* and *Sinostoma* display rather plesiomorphic genitalic characters (Martens 2016, 2017) and may be basally derived members of the nemastomatine radiation. They will probably be placed at or near the base of the still incomplete molecular genetic tree (Schönhofer and Martens 2012).

## Supplementary Material

XML Treatment for Starengovia

XML Treatment for Starengovia quadrituberculata
